# On the instability of syzygy bundles on toric surfaces

**DOI:** 10.1007/s10231-025-01604-w

**Published:** 2025-09-06

**Authors:** Lucie Devey, Milena Hering, Katharina Jochemko, Hendrik Süß

**Affiliations:** 1 School of Mathematics, The University of Edinburgh, Edinburgh, UK; 2 KTH Royal Institute of Technology, Stockholm, Sweden; 3 Institut für Mathematik, Friedrich-Schiller-Universität Jena, Jena, Germany

**Keywords:** Vector bundles, Stability, Syzygy bundles, Toric varieties, 14J60, 14M25, 14J26, 14D20

## Abstract

We show that for every toric surface *X* apart from $\mathbb {P}^2$ and $\mathbb {P}^1\times \mathbb {P}^1$ and every ample line bundle $\mathcal {L}$ on *X* there exists an ample polarisation *A* for *X*, such that the syzygy bundle $M_{\mathcal {L}^{\otimes d}}$ associated to the tensor power $\mathcal {L}^{\otimes d}$ is not stable with respect to *A* for every *d* sufficiently large.

Let *X* be a projective variety of dimension *n* over an algebraically closed field *k* and let $\mathcal {L}$ be an ample and globally generated line bundle on *X*. The *syzygy bundle* $M_{\mathcal {L}}$ is defined by the following short exact sequence:

1 $$\begin{aligned} 0 \rightarrow M_{\mathcal {L}} \rightarrow H^0(X,\mathcal {L})\otimes \mathcal {O}_X \rightarrow \mathcal {L}\rightarrow 0. \end{aligned}$$

There has been interest in (slope) stability of syzygy bundles as they come up in the study of defining equations and higher syzygies of embeddings of algebraic varieties, and there also are connections to tight closure. The study of syzygy bundles goes back to Butler [1]. On curves, $M_L$ is stable as soon as $\deg L\ge g+1$, and several cases are known in higher dimensions, see for example the introduction to Ein et al. [4] with more recent advances in Torres-López [7, 8]. More recently, Rekuski [6] showed that for an ample and globally generated line bundle $\mathcal {L}$ on a smooth projective variety, $M_{\mathcal {L}^d}$ is stable with respect to $\mathcal {L}$ for $d\gg 0$ and provides effective bounds for *d*, generalising a result on surfaces by Ein et al. [4].

In [4, Problem 2.5], the authors ask whether for $\mathcal {L}$ ample and *A* an arbitrary ample divisor, $M_{\mathcal {L}^d}$ is slope stable with respect to *A* for $d\gg 0$. The following theorem shows that this is not the case.

## Theorem 1

Let *X* be a smooth toric surface not isomorphic to $\mathbb {P}^2$ and $\mathbb {P}^1\times \mathbb {P}^1$. Then for every ample divisor *D* there exists an ample polarisation *A* such that $M_{O(dD)}$ is not stable with respect to *A* for $d\gg 0$.

For an ample divisor *D* on a Hirzebruch surface, we can precisely describe the set of ample polarizations *A* and $d>0$ such that $M_{\mathcal {O}(dD)}$ is not stable with respect to *A*. Let $\mathbb {F}_{\ell } \rightarrow \mathbb {P}^1$ be the $\ell$-th Hirzebruch surface with *F* being a fiber and *S* the unique section with self-intersection $-\ell$.

## Theorem 2

Let $\mathbb {F}_{\ell }$ be a Hirzebruch surface with $\ell \ge 1$ and $A=A_1S+A_2F$ and $D=B_1S+B_2F$ be ample divisors on $\mathbb {F}_{\ell }$. Set $a=A_2/A_1$ and $b=B_2/B_1$. If

2 $$\begin{aligned} a> &   \frac{2b(b-\ell )}{\ell } +\ell \text { and } d> \frac{2b-2ab+a\ell + 2b\ell -\ell ^2}{B_1(a\ell -2b(b-\ell )-\ell ^2)} \text { or } \end{aligned}$$

3 $$\begin{aligned} a= &   \frac{2b(b-\ell )}{\ell } +\ell \text { and } b> \frac{3\ell }{4} + \sqrt{\frac{\ell ^2}{16}+\frac{\ell }{2}} \text { and } d>0 \text { or }\end{aligned}$$

4 $$\begin{aligned} a< &   \frac{2b(b-\ell )}{\ell } +\ell \text { and } d< \frac{2b-2ab+a\ell +2b\ell -\ell ^2}{B_1(a\ell -2b(b-\ell )-\ell ^2)}. \end{aligned}$$

Then $M_{\mathcal {O}(dD)}$ is not stable with respect to *A*. A destabilising subbundle is given by $M_{\mathcal {O}(dD-S)}$.

## Example 3

Let $X=\mathbb {F}_{1}$ be the blow up of the projective plane at one point, $S=E$ the exceptional divisor and *H* the hyperplane divisor. Then $F=H-E$. For $D =5S+6F$ and $d > 17$ we have that $M_{\mathcal {O}(dD)}$ is not semistable with respect to $A=-K_X = 2S+3F$. The destabilising subbundle is $M_{\mathcal {O}(dD-S)}$.

In a forthcoming paper, by exploiting the fact that *X* is toric, we will show that the bundle $M_{\mathcal {O}(D)}$ from Example 3 is in fact stable. Hence, this provides an example of an ample line bundle $\mathcal {L}=\mathcal {O}(D)$ and a polarization *A* where $M_\mathcal {L}$ is *A*-stable, but $M_{\mathcal {L}^{\otimes d}}$ is unstable with respect to *A* for $d\gg 0$.

We will see that the destabilizing subbundles are kernel bundles associated to $D'$, where $D'$ is movable, and $D-D'$ is effective. In a forthcoming article we will show that on a toric variety the subbundle of maximal slope of $M_{\mathcal {O}(D)}$ for *D* ample is of this form. Generalised versions of these subbundles appeared e.g. already in [1, 1.9].

## Proofs of the theorems

Recall that the slope of a reflexive sheaf $\mathcal {F}$ with respect to a polarisation given by an ample divisor *A* is given by

$$\begin{aligned} \mu _A(\mathcal {F})=\frac{c_1(\mathcal {F})\cdot A^{n-1}}{\textrm{rk}({\mathcal {F}})}.\end{aligned}$$

We call a vector bundle $\mathcal {E}$ stable (semistable) with respect to *A* if for every reflexive subsheaf $\mathcal {F}$ of $\mathcal {E}$, we have $\mu _A(\mathcal {F})<\mu _A(\mathcal {E})$ ($\mu _A(\mathcal {F})\le \mu _A(\mathcal {E})$).

Now let $\mathcal {L} = O(D)$ for some ample divisor *D*. Then it follows from the short exact sequence (1) and additivity of first chern classes that $c_1(M_{\mathcal {O}(D)}) =-D$. So

5 $$\begin{aligned} \mu _A(M_{\mathcal {O}(D)}) = \frac{-D\cdot A^{n-1}}{h^0(X,D)-1}. \end{aligned}$$

### Lemma 4

Let $D, D'$ be divisors such that $D-D'$ is effective and $\mathcal {O}(D), \mathcal {O}(D')$ are globally generated. Then $M_{\mathcal {O}(D')}$ is naturally a subbundle of $M_{\mathcal {O}(D)}$.

### Proof

This follows from the following diagram.Indeed, the square on the right commutes, which implies the claim. $\square$

### Proposition 5

Let *X* be a smooth projective surface and *D* and *A* ample divisors. Let *S* be an effective divisor and *d* be a positive integer large enough such that $\mathcal {O}(dD)$ and $\mathcal {O}(dD-S)$ are globally generated.

1. If $(S\cdot A)(D^2) - 2(D\cdot A)(D\cdot S)>0$, then $\mu _A\left( M_{\mathcal {O}(dD-S)}\right) > \mu _A\left( M_{\mathcal {O}(dD)}\right)$ for $d\gg 0$.
2. Assume in addition that *X* is rational and that the higher cohomology of $\mathcal {O}(dD)$ and $\mathcal {O}(dD-S)$ vanishes. Then $\mu _A\left( M_{\mathcal {O}(dD-S)}\right) > \mu _A\left( M_{\mathcal {O}(dD)}\right)$ if and only if
  6 $$\begin{aligned} (S\cdot A)(D^2) - 2(D\cdot A)(D\cdot S)>0&\text { and }&d> \frac{-(D\cdot A)(S^2+S\cdot K_X) +(S\cdot A)(D\cdot K_X)}{(S\cdot A)(D^2) - 2(D\cdot A)(D\cdot S)} \text { or } \end{aligned}$$
  7 $$\begin{aligned} (S\cdot A)(D^2) - 2(D\cdot A)(D\cdot S) = 0&\text { and }&(D\cdot A)(S^2+S\cdot K_X)-(S\cdot A)(D\cdot K_X) >0 \end{aligned}$$
  8 $$\begin{aligned}  &   \text { and } d>0 \text { or } \nonumber \\ (S\cdot A)(D^2) - 2(D\cdot A)(D\cdot S)< 0&\text { and }&d < \frac{-(D\cdot A)(S^2+S\cdot K_X)+(S\cdot A)(D\cdot K_X)}{(S\cdot A)(D^2) - 2(D\cdot A)(D\cdot S) }. \nonumber \\ \end{aligned}$$
  Moreover, $\mu _A\left( M_{\mathcal {O}(dD-S)}\right) = \mu _A\left( M_{\mathcal {O}(dD)}\right)$ if and only if
  $$\begin{aligned}  &   (S\cdot A)(D^2) - 2(D\cdot A)(D\cdot S)\ne 0 \text { and }\\  &   d=\frac{-(D\cdot A)(S^2+S\cdot K_X) +(S\cdot A)(D\cdot K_X)}{(S\cdot A)(D^2) - 2(D\cdot A)(D\cdot S)} \text { or } \\  &   (S\cdot A)(D^2) - 2(D\cdot A)(D\cdot S)=0 \text { and } \\    &   (D\cdot A)(S^2+S\cdot K_X)-(S\cdot A)(D\cdot K_X) =0 \text { and }d>0. \end{aligned}$$

### Proof

Set $\mathcal {L}=\mathcal {O}(dD)$, $\mathcal {L}_1=\mathcal {O}(dD-S)$. Let $p=p_a(X)$ denote the arithmetic genus of *X*. For (1) we pick *d* sufficiently large so that the higher cohomology of $\mathcal {L}$ and $\mathcal {L}_1$ vanishes and for (2) it vanishes by assumption. Then Riemann-Roch implies

$$\begin{aligned}  &   h^0(\mathcal {L})=\chi (\mathcal {L})=1+p+\frac{(dD)^2-(dD)\cdot K_X}{2} \text { and }\\  &   h^0(\mathcal {L}_1)=1+p+\frac{(dD-S)^2-(dD-S)\cdot K_X}{2}.\end{aligned}$$

We consider the subbundle $M_{\mathcal {L}_1} \subset M_\mathcal {L}$. Using this notation we obtain from (5)

$$\begin{aligned} \mu _A(M_\mathcal {L})&=-\frac{dD \cdot A}{h^0(\mathcal {L})-1}= -\frac{dD \cdot A}{p+\frac{1}{2}\left( d^2D^2 - dD \cdot K_X\right) }\\ \mu _A(M_{\mathcal {L}_1})&=-\frac{(dD-S) \cdot A}{h^0(\mathcal {L}_1)-1}\\&=- \frac{dD\cdot A- S\cdot A}{p+\frac{1}{2}\left( d^2 D^2 - 2dD\cdot S + S^2 - (dD\cdot K_X - S\cdot K_X\right) }. \end{aligned}$$

We want to determine under what conditions we have $\mu _A(M_{\mathcal {L}_1})> \mu _A(M_\mathcal {L})$. We write $\mu _A(M_{\mathcal {L}_1}) - \mu _A(M_\mathcal {L})$ as a fraction with common denominator $2(h^0(\mathcal {L})-1)(h^0(\mathcal {L}_1)-1)$, which is clearly positive. The numerator of $\mu _A(M_{\mathcal {L}_1}) - \mu _A(M_\mathcal {L})$ is

$$\begin{aligned} \left( (S\cdot A)(D^2)- 2(D\cdot A)(D\cdot S) \right) d^2 + ((D\cdot A)(S^2+S\cdot K_X)-(S\cdot A)(D\cdot K_X))d +2 (S\cdot A)p. \end{aligned}$$

For (1) note that for $d\gg 0$, the quadratic term dominates, so if $(S\cdot A)(D^2)- 2(D\cdot A)(D\cdot S)>0$, we have $\mu _A(M_{\mathcal {L}_1}) - \mu _A(M_\mathcal {L})>0$.

For (2), we have $p=0$, so as $d>0$, we can divide by *d*, and we need to determine under what conditions

9 $$\begin{aligned} \left( (S\cdot A)(D^2)- 2(D\cdot A)(D\cdot S) \right) d + ((D\cdot A)(S^2+S\cdot K_X)-(S\cdot A)(D\cdot K_X))>0.\nonumber \\\end{aligned}$$

Inspecting the three cases $(S\cdot A)(D^2) > 2(D\cdot A)(D\cdot S), (S\cdot A)(D^2) = 2(D\cdot A)(D\cdot S),$ and $(S\cdot A)(D^2) < 2(D\cdot A)(D\cdot S)$, we obtain the result.

The slopes are equal if and only if the left hand side of (9) is zero, which implies the statement. $\square$

Let us consider the Hirzebruch surface of degree $\ell$ that is $\mathbb {F}_\ell =\operatorname {Proj}_{\mathbb {P}^1}(\mathcal {O}_{\mathbb {P}^1} \oplus \mathcal {O}_{\mathbb {P}^1}(\ell ))$. There is the structure morphism $\mathbb {F}_\ell \rightarrow \mathbb {P}^1$. Let $S\subset \mathbb {F}_\ell$ denote the unique section of self intersection $-\ell$ and let *F* be a fibre. Then the Picard group and effective cone are generated by *S* and *F*. The intersection product is given by $S^2=-\ell$, $F^2=0$ and $S\cdot F=1$. The canonical divisor is given by $K_{\mathbb {F}_\ell }=-2S-(2+\ell )F$.

### Proof of Theorem 2

Note that $B_1S + B_2F$ is nef if and only if $B_1\ge 0$ and $B_2\ge \ell B_1$ and ample if and only if strict inequalities hold. In particular, if *D* is ample, then *dD* and $dD-S$ are nef for all $d\ge 1$. Since a nef bundle on a toric variety is globally generated it follows that $\mathcal {O}(dD)$ and $\mathcal {O}(dD-S)$ are globally generated. By Lemma 4, $M_{\mathcal {O}(dD-S)} \subset M_{\mathcal {O}(dD)}$ is a subbundle.

We now apply the comparison of slopes from Proposition 5(2) to determine when $M_{\mathcal {O}(dD-S)}$ is a destabilising subbundle of $M_{\mathcal {O}(dD)}$. It is well-known that the higher cohomology of nef line bundles on toric varieties vanishes, so the higher cohomology of $\mathcal {O}(dD)$ and $\mathcal {O}(dD-S)$ vanishes.

Let $A' = S+aF$ and $D'=S+bF$. Then we calculate.

$$\begin{aligned}&(A\cdot S)(D^2)-2(D\cdot A)(D\cdot S) =(A_1A'\cdot S)((B_1D')^2)-2(B_1D'\cdot A_1A')(B_1D'\cdot S) \\&=A_1B_1^2(a\ell -2b(b-\ell ) -\ell ^2) \text { and } \\&-(D\cdot A)(S^2+S\cdot K_X)+(S\cdot A)(D\cdot K_X)=A_1B_1(2b-2ab+a\ell + 2b\ell -\ell ^2) \end{aligned}$$

The sign of the first expression determines which of the cases in Proposition 5(2) we are in. On the other hand, since $A_1, B_1$ are positive integers its sign coincides with the sign of

$$\begin{aligned} a - \frac{2b(b-\ell )}{\ell } - \ell . \end{aligned}$$

In the cases where subsituting

$$\begin{aligned}\frac{-(D\cdot A)(S^2+S\cdot K_X) +(S\cdot A)(D\cdot K_X)}{(S\cdot A)(D^2) - 2(D\cdot A)(D\cdot S)} = \frac{2b-2ab+a\ell + 2b\ell -\ell ^2}{B_1(a\ell -2b(b-\ell )-\ell ^2)} \end{aligned}$$

in (6) and (8) of Proposition 5(2) gives (2) and (4) in Theorem 2, respectively.

Now, if $a - \frac{2b(b-\ell )}{\ell } - \ell = 0$ then (7) in Proposition 5 has to hold. This leads to

$$\begin{aligned} A_1B_1(a(\ell -2b) + 2b+2b\ell - \ell ^2) > 0. \end{aligned}$$

Dividing by $A_1B_1$ and substituting *a* by $\frac{2b(b-\ell )}{\ell } +\ell$ gives $\frac{4b}{\ell } \left( -b^2+\frac{3}{2} \ell b- \frac{1}{2}\left( \ell ^2-\ell \right) )\right) >0$ which is equivalent to $b^{2} - \frac{3}{2}\ell b + \frac{1}{2}(\ell ^{2}-\ell ) < 0$.

The two roots of the polynomial on the left-hand-side are

$$\begin{aligned}\frac{3\ell }{4} \pm \sqrt{\frac{\ell ^2}{16}+\frac{\ell }{2}}\end{aligned}$$

Note that since *D* is ample, we must have $b>\ell$ and the smaller root is in fact smaller than $\ell$. Now Proposition 5 (7) implies that when $a = \frac{2b(b-\ell )}{\ell } +\ell$ and

$$\begin{aligned} \ell< b < \frac{3\ell }{4} + \sqrt{\frac{\ell ^2}{16}+\frac{\ell }{2}} \end{aligned}$$

the syzygy bundle $M_{\mathcal {O}(dD)}$ is unstable for all $d>0$.

### Example 6

As in Example 3 we consider again $\mathbb {F}_1$ and stability with respect to $-K_{\mathbb {F}_1}$. For $\ell =1$ condition (2) becomes $a = \frac{3}{2} > 2b(b-1)+1$ and $d> \frac{2b+1}{B_1(1-4b(b-1))}$. For example, if $b = \frac{9}{8}$, then the syzygy bundle $M_{\mathcal {L}^{\otimes d}}$ with $\mathcal {L}=\mathcal {O}(8S+9F)$ is $(-K_{\mathbb {F}_1})$-unstable for $d \ge 1$. Indeed, we have

$$\begin{aligned} a=\frac{3}{2} = \frac{96}{64} > 2\cdot \frac{9}{8}\cdot \frac{1}{8}+1 =\frac{82}{64}=2b(b-1)+1 \text { and } \frac{2b+1}{B_1(1-4b(b-1))} = \frac{13}{14}, \end{aligned}$$

so Theorem 2 implies that $M_{\mathcal {L}^d}$ is $(-K_{\mathbb {F}_1})$-unstable for $d\ge 1$ with destabilising subbundle $M_{\mathcal {O}(7S+9F)}$.

To obtain a necessary condition when a tensor power of $\mathcal {L}=\mathcal {O}(S+bF)$ can have $(-K_{\mathbb {F}_1})$-stable syzygy bundle, we solve condition  (4) in *b* for $\ell =1$ and $a=\frac{3}{2}$, which leads to $b \ge \frac{1}{2}(\sqrt{2} +1)$. Note that this is in line with [6, Theorem 4.4], since the sufficient condition there is more restrictive than the necessary condition given above.

### Remark 7

It is natural to ask whether in the setting of Theorem 2 for $d \gg 0$ the bundle $M_{\mathcal {O}(dD)}$ is in fact *A*-semistable if the conditions of Theorem 2 are not fulfilled. In a forthcoming paper we will give a combinatorial proof that this is true.

### Proposition 8

Let *X* be a smooth surface of Picard number at least 3 having an effective cone generated by curves $E_1, \ldots , E_m$ with mutual intersection multiplicity of at most 1. Then for every ample divisor *D* there exists an ample polarisation *A* such that for $d\gg 0$ the syzygy bundle $M_{O(dD)}$ is not stable with respect to *A*.

### Proof

We consider $E_j$ with the property that $D\cdot E_j$ is minimal among the $E_i$. Let $t=\max \{t \mid D -tE_j \text { is nef}\}$ and set $A=D-tE_j$. Since *A* is on the boundary of the nef cone, we have $A\cdot E_i=0$ for some generator $E_i$ and for all such $E_i$ one can deduce from the assumptions $E_i\cdot E_j \in \{0,1\}$ and *D* ample that $D\cdot E_i=t$. By the choice of $E_j$ this implies

10 $$\begin{aligned} t \ge D \cdot E_j. \end{aligned}$$

Now, we look at the condition of Proposition 5(1) where we set $S=E_j$. By [3, Prop. 2.3.], we may assume that the curves $E_1, \ldots , E_m$ have negative self-intersection. We then obtain

$$\begin{aligned} (S\cdot A)D^2 - 2(D\cdot A)(D\cdot S)&= (E_j\cdot (D-tE_j))D^2 - 2(D\cdot (D-tE_j))(D\cdot E_j)\\&= - t(E_j^2)D^2 + 2t (D \cdot E_j)^2 - D^2(D\cdot E_j) \\&> - D^2(D\cdot E_j) - tE_j^2D^2\\&\ge - D^2(D\cdot E_j) + tD^2\\&= D^2(t - D\cdot E_j)\\&\ge 0. \end{aligned}$$

Where the last inequality follows from (10). Note, that *A* is only nef, not ample. However, replacing *A* by $A'=A+\epsilon E_j$ will be ample for $0 < \epsilon \le 1$ and by continiuty we have $2(D\cdot A')(D\cdot S) - (S\cdot A')D^2 < 0$ for $\epsilon$ sufficiently small. Now pick *d* sufficiently large so that $\mathcal {O}(dD)$ and $\mathcal {O}(dD -S)$ are globally generated. Then Lemma 4 implies that $M_{\mathcal {O}(dD -S)}$ is a subbundle of $M_{\mathcal {O}(dD)}$ and Proposition 5 implies that for $d\gg 0$ it is destabilising. $\square$

### Proof of Theorem 1

The case of Hirzebruch surfaces has been dealt with in Theorem 2, so assume that *X* is not a Hirzebruch surface, $\mathbb {P}^1 \times \mathbb {P}^1$ or $\mathbb {P}^2$. It follows from the classification of toric surfaces [5, p.43], that in this case *X* has Picard rank at least 3. The effective cone of any toric variety is generated by the classes of the (finitely many) torus invariant divisors [2, Lemma 15.1.8.], and on a smooth toric surface the intersection numbers of two torus invariant divisors is either zero or one [5, p.98]. So the conditions of Proposition 8 are fulfilled.

### Remark 9

The conditions on *X* in Proposition 8 are also fulfilled for various non-toric surfaces e.g. for del Pezzo surfaces of degree at least 3 or the blowup of $\mathbb {P}^2$ in an arbitrary number of points on a line. Hence, for those surfaces the statement considered in [4, Problem 2.5] is also violated for every ample line bundle $\mathcal {L}$.
